# Adding to the knowledge on Patient and Public Involvement: Reflections from an experience of co‐research with carers of people with dementia

**DOI:** 10.1111/hex.13049

**Published:** 2020-03-17

**Authors:** Claudio Di Lorito, Maureen Godfrey, Marianne Dunlop, Alessandro Bosco, Kristian Pollock, Veronika van der Wardt, Rowan H. Harwood

**Affiliations:** ^1^ Division of Rehabilitation, Ageing and Wellbeing School of Medicine University of Nottingham Queen's Medical Centre Nottingham UK; ^2^ Division of Psychiatry and Applied psychology School of Medicine University of Nottingham Nottingham UK; ^3^ School of Health Sciences Queen's Medical Centre University of Nottingham Nottingham UK; ^4^ WissenschaftlicheMitarbeiterin ZentrumfürMethodenwissenschaften und GesundheitsforschungAbteilungfürAllgemeinmedizin Präventive und Rehabilitative Medizin Philipps‐Universität Marburg Marburg Deutschland

**Keywords:** carers, co‐research, dementia, patient and public involvement (PPI), qualitative research

## Abstract

**Background:**

Patient and Public Involvement (PPI) in research ensures that publicly funded research reflects the priorities of the people who will be affected by its results. Co‐research, a branch of PPI, is equal partnership between academic researchers and members of the public, who steer and conduct research together.

**Objectives:**

To propose a model for good practice in co‐researching with carers of people with dementia, by reporting and synthesizing the personal reflections of the academic and lay researchers around the methodological issues, benefits, and challenges of co‐research.

**Design:**

An academic researcher and two lay researchers with lived experience of caring with someone with dementia collaborated in all stages of a qualitative research study, including development of the research protocol and topic guide, data collection, analysis and synthesis, and dissemination of findings. Throughout the study, the academic and lay researchers annotated reflections of their experience in personal diaries. Data from the diaries were synthesized and mapped out in a model for good practice in co‐research.

**Results:**

Co‐research yielded benefits for all those involved and on research outputs. There were practicalities and challenges that required extra resources, in order to make the involvement of lay researchers meaningful and effective.

**Discussion:**

The model for good practice illustrates overarching and stage‐specific guidelines, which can inform researchers and members of the public wishing to undertake good practice in co‐research.

## 1 INTRODUCTION

Patient and Public Involvement (PPI) is involvement in research of members of the public who have lived experience of the phenomenon under investigation. In PPI, research is carried out ‘with’ or ‘by’ members of the public, as opposed to ‘to’, ‘about’, and ‘for’ them.^1^ PPI is grounded in the principle of ‘knowledge by experience’, as opposed to ‘knowledge by expertise’, which deconstructs the notion of hierarchical knowledge, passed by academic experts to lay members.^2^, ^3^ Knowledge, through a PPI perspective, is co‐built by ‘experts by training’ and ‘experts by experience’.^4^ PPI has gained momentum in several countries and many funders, such as the United Kingdom National Institute of Health Research (NIHR) require that PPI plans are included in social and health care research grants.

There are different forms of PPI.^5^ These range from consultation, where members of the public provide research input (eg evaluating research proposals) through inclusion in advisory committees, to leading the whole research cycle by members of the public.^6^ Collaboration, or co‐research, lies in the middle of the ‘spectrum’, and indicates equal partnership between academic researchers and members of the public, who steer and conduct research together.^6^ Ideally, co‐research occurs in all stages of the research cycle, including the development of study design and questions, research materials, data collection and analysis, and report and dissemination of findings.^7^

In dementia research, lay researchers can be people living with the condition who have the ability to undertake a research role, as well as carer (henceforth defined ‘carers’).^6^ Co‐research enables people with lived experience of dementia to have a voice in identifying research priorities and direct the research process.^8^ This responds to the call for research to be qualified, ethical, and relevant to the primary stakeholders.^9^ Co‐research is also concerned with equitability (ie redistributing power in the research process) and challenges the power in the hands of the experts‐by‐training, typical of academic‐led research.^10^

Compared to other vulnerable populations, such as people with Intellectual Disability,^11^ co‐research in dementia is still limited practice. However, recent publications have evidenced benefits for academic researchers, research participants, lay researchers with dementia, and the public.^8^, ^12^

There is a growing emphasis on co‐research in dementia, as demonstrated by the recent release of a special issue on the topic in the journal ‘Dementia’. However, there is currently only a handful of empirical studies based on co‐research with carers of people with dementia^13^, ^14^, ^15^ and only a couple have reported on methodological issues.^16^, ^17^ Given the challenges of involving people with deteriorating cognition in research and that the method of co‐research is still in its evolutionary stage, it is timely to experiment with different types of collaborators with lived experience of dementia. It is also crucial to recognize the central role that carers have in the production and delivery of care^18^ and the burden that a caring role may generate,^19^ to the point that carers have been defined ‘the invisible patients’.^20^ This renders carers experts‐by‐experience and requires that their voices are represented in research that affects their lives.

The present study was co‐produced with PPI members with lived experience of dementia. It is ethically grounded in the Alzheimer Europe Strategic Plan 2016‐2020,^21^ and the Prime Minister's second Challenge on Dementia,^22^ aiming to ensure partnership in research, policy development, and service design between researchers, people with dementia and their carers, funders, and society. It aims to propose a model for good practice in co‐researching with carers of people with dementia, by reporting and synthesizing the personal reflections of the academic and lay researchers around the methodological issues, benefits, and challenges of an experiment of co‐research within the context of a large randomized controlled trial (RCT).

## 2 METHODS

**Figure 1:**
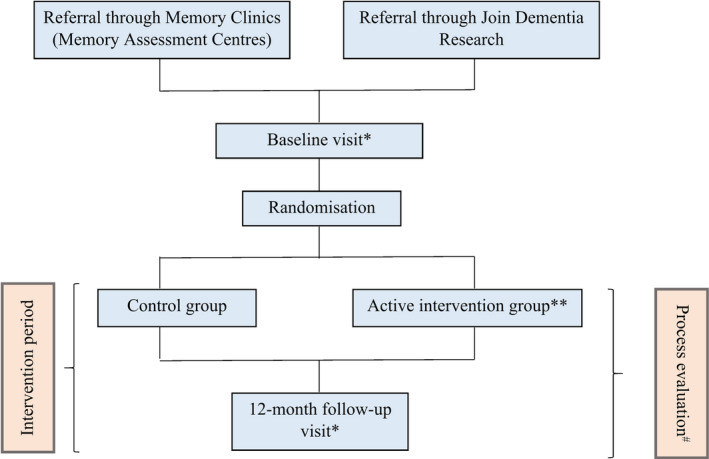
PrAISED activities. *Includes informant and participant‐reported measures on sociodemographics, medical history, medications, frailty, mobility, personality, cognition, quality of living, health, disability, falls efficacy, mood/ affect, activities of daily living, muscle strength, physical activity, static and dynamic balance, carer strain, and carer's health. **Receives physical exercises, functional activities (eg shopping), physical activity promotion; risk enablement; environmental assessment, community engagement and provision of information. ^#^Includes qualitative interviews with participants with dementia and their carers at month six ad 12 of the intervention

This study adheres to the consolidated criteria for reporting qualitative research (COREQ) checklist (Appendix 1).^23^ It is based on an experiment of co‐research with carers of people with dementia embedded in the Promoting Activity, Independence and Stability in Early Dementia (PrAISED) RCT. The PrAISED was designed to promote activity and independence among people with mild dementia or mild cognitive impairment (Figure 1).^24^ Embedded in the PPI of PrAISED, co‐research occurred in the context of the process evaluation,^25^ a sub‐study aiming to investigate the experience of the participants with dementia and their carers (Figure 1).

### 2.1 Setting

This section describes how co‐research was set up and carried out throughout the stages of the research cycle identified by Mockford et al.^17^

#### 2.1.1 Thinking and planning

The experiment of co‐research was not initially considered/ costed in the PrAISED proposal/protocol. When the main author (CDL) joined PrAISED in 2018 to lead on the process evaluation package, having previous experience of co‐researching, he proposed to embed an experiment of co‐research in the process evaluation package. CDL was able to utilize the financial and staff resources of the PrAISED RCT to set up and carry out co‐research.

The co‐research team was made up of an academic researcher and two lay researchers. The academic researcher was a Research Fellow from the PrAISED team with expertise in qualitative research (CDL, referred to as ‘academic researcher’). The lay researchers were two PPI collaborators ((MG and MD, referred to as ‘lay researchers’) with lived experience of caring for someone with dementia. The lay researchers had previously collaborated in the PrAISED RCT study design (eg designing the therapy programme and developing research documents) and study management. Both PPI representatives sat on the Trial Management Committee and one (MG) was also a study co‐applicant.

Given their previous PPI roles at the University of Nottingham, their knowledge and expertise in PrAISED, their different background and skillset, their experience in dementia care and research, and their personal aspirations, the lay researchers were identified as the most suitable collaborators for co‐research. No formal recruitment was undertaken. The academic researcher approached the lay researchers with a proposal and the lay researchers accepted the invitation to collaborate. While the academic researcher had publications around co‐research^11^, ^12^ and the lay researchers had taken on previous PPI roles, none had previous experience of co‐researching.

The co‐researchers’ role was not pre‐imposed by the academic team, but it was co‐designed by the lay and academic researchers through discussions around the tasks that the lay researchers felt most comfortable undertaking. It was agreed that they would be involved in all stages of the process evaluation research cycle, including designing the study protocol, developing the topic guide, collecting and analysing data, and disseminating research findings. This would ensure a genuine partnership in areas of research (eg data analysis) which have been traditionally ‘academia‐dominated’.^26^ Being one of the co‐applicants in the PrAISED RCT, lay researcher MG was involved in budget planning allocated for PPI contribution, which was based on INVOLVE guidelines.^27^ It was agreed that the lay researchers would be compensated for their time spent working on the process evaluation in any of the stages of the study.

In relation to ethics, while the approval received for the PrAISED RCT covered the process evaluation sub‐study too, we were unsure whether involvement of PPI members as lay researchers required further ethics approval. As prescribed in the Alzheimer Europe's pamphlet on PPI,^8^ the academic team liaised with the Head of Volunteering at the University of Nottingham for advice. Governance advice from the sponsor was that the lay researchers would not need further ethics approval, as they would see participants together with the academic researcher. The experiment of co‐research was therefore part of the ethics approval received by the PrAISED study (Ref.: 18/YH/0059. ISRCTN Registration Number: 15320670).

In relation to protocol design, the main author of the manuscript (CDL) developed a draft, which was passed to all co‐authors (including the lay researchers) to comment and provide feedback on content and language. The lay researchers’ input was crucial in ensuring that the study was empowering for the participants with dementia. For example, although it was initially envisioned that the qualitative interviews would be conducted with the carers and the participants with dementia separately, the lay researchers suggested that asking to the participant with dementia about their preference would be a more ethical approach.

The development of the interview schedule also involved a collaborative effort. The topic guide consisted of open‐ended questions, designed to stimulate reflection in the participants about the factors that had contributed to their experience in the PrAISED RCT (Appendix 2). The academic researcher developed a tentative version of the topic guide, which was then discussed and edited in a meeting with the lay researchers. This meeting ensured that the interview questions were relevant, meaningful, and jargon‐free for participants with dementia. It was found that the terminology and structure of some of the questions was difficult to understand. For example, the concept of control was deemed to be too abstract. The question ‘How much control do you feel you had in developing the programme of physical activity?’ was therefore changed into ‘How much were you able to decide what to do in PrAISED?’

#### 2.1.2 Preparing

As per the NIHR guidance on co‐producing a research project,^28^ some preparatory training sessions were undertaken to enable the lay researchers to undertake their role in co‐research.^8^

The needs of lay researchers were carefully evaluated and discussed. The lay researchers were asked to think about the role and reflect on which aspects they might find challenging and which specific skills they believed they should develop/boost. Having been PPI members in various research projects in the University of Nottingham, the lay researchers felt confident to fulfil most of the duties that their role included. The lay researchers had previously been trained in data analysis, through a two‐day course delivered by the University of Nottingham research staff. The training consisted in an introductory session and workshop on qualitative data analysis. The course participants were then asked to work independently on the coding of a sampled interview excerpt before the second session. The homework was discussed in a group format in the second workshop. The lay researchers felt that, given the vulnerability of the target population, training to carry out the qualitative interviews with participants was required. A plan was then set up, which comprised a half‐day session held by the academic researcher and attended by both lay researchers.

In the first hour of the session, the academic researcher introduced the concept of process evaluation and the scope of the study in a jargon‐free language and appropriate format, and then responded to the questions that the lay researchers might have. The following 2 hours were focused on training. It was agreed that the aim of the training would be to make the lay researchers confidence enough to be involved in the interviewing process in such a way that it produced benefits for themselves, the participants, and data collection. It was agreed that the academic researcher, who had expertise in qualitatively interviewing vulnerable populations would provide refreshers around interviewing techniques, using the interview schedule, managing interactions, non‐verbal communication, sensitive topics, anonymity, and confidentiality.

In the remainder of the training session, the co‐research team arranged the practicalities of the interviews (eg transport, dates). In accordance with good practice in co‐research,^8^, ^28^ ground rules, and specific research tasks were established, based on their suitability to the lay researchers’ skills and aspirations and to research needs. The themes in the interview topic guide were shared between the academic and lay researchers. It was decided that, based on the concept of ‘expert‐by‐training’ and ‘expert‐by‐experience’, the academic researcher (with a psychology background) would ask questions about personal beliefs and motivation, while the lay researchers would investigate issues related to quality of life, such as emotional support and independence (Appendix 2).

This session was also an opportunity to build up rapport between team members. Maintenance of an open, honest, and trusting relationship throughout (and beyond) the collaboration was key to successful co‐research. For example, the team created informal safe spaces out of the research environment (eg meetings in the lay researchers’ homes) to further develop the quality relationships and make the meetings more accessible.^29^ The team agreed that the training process would be iterative throughout the study, based on the emerging needs of the lay researchers. This proved effective after the lay researchers experienced a moral imperative to offer advice to participants (eg signpost activities available in the community) who exhibited difficulties (eg social isolation) during the interviews. In line with Corbin and Morse,^30^ the co‐research team felt a moral obligation to pass on information which might help alleviate participants’ emotional burden. However, In order to avoid breaching boundaries between the scope of the interview and the intervention, and to avoid a negative impact on data integrity, information was provided after the interview session had ended, when appropriate (ie a decision was made by the academic team on the type of information that would not have an impact on the trial and sent to the participants by mail).

#### 2.1.3 Gathering

The participants with dementia in the PrAISED process evaluation and their respective identified carer were purposively selected by the academic researcher. This ensured that the sample was representative of the PrAISED RCT participants in terms of gender, ethnicity, relationship status, geographical location, and adherence to the exercise programme (ie low and high adherence).

The academic researcher contacted the participants through phone to enquire on their availability to take part in the process evaluation interviews and to confirm whether the participants were happy to be interviewed through co‐research. None refused. Once they had accepted to participate, the participants received a letter with the details of the appointment and an information sheet. The participants and their carers were interviewed (as dyads) through qualitative semi‐structured interviews in their private home. The academic researcher carried out half the number of the interviews alone (ie with no layresearcher). It was agreed with the lay researchers that gathering interview data through two different configurations (ie with and without the layresearcher) would ensure easier identification of the added benefits and challenges of co‐research.

In regards to the co‐research interviews, the academic researcher and the lay researcher (one per interview) interviewed travelled to the participants’ homes together and used the time before and after the session to brief and debrief. The debriefing was an opportunity to discuss any aspects of the interview session that might have had a negative impact on the emotional wellbeing of the lay researchers. Verbal information (eg access to counselling services) and advice (eg how to process negative accounts from participants) were provided, when needed.

On the day of the interview, before the session commenced, each of the co‐research team members introduced themselves. Alternatively, the academic and layresearcher answered any questions that the participants might have, and gathered consent. The interviews were audio‐recorded and continued until data saturation was achieved. The interviews were carried out from April to August 2019. In total, the co‐research team interviewed seven participants with dementia and their carers (14 people in total). Carer participants were five spouses, one sibling, and one child. The sessions lasted on average 1 hour.

#### 2.1.4 Analysing

A professional agency transcribed the interviews verbatim. The transcripts were not returned to participants for comments. The transcripts were transferred onto NVivo 12.^31^

The lay researchers decided to which extent they felt comfortable being involved in data analysis and the academic team agreed on an analysis plan. The academic researcher analysed independently (ie without the lay researchers) the transcripts of the interview he had carried out alone. Again, it was agreed that comparing different configurations in data analysis (ie with and without lay researchers) would facilitate learning points.

**Figure 2:**
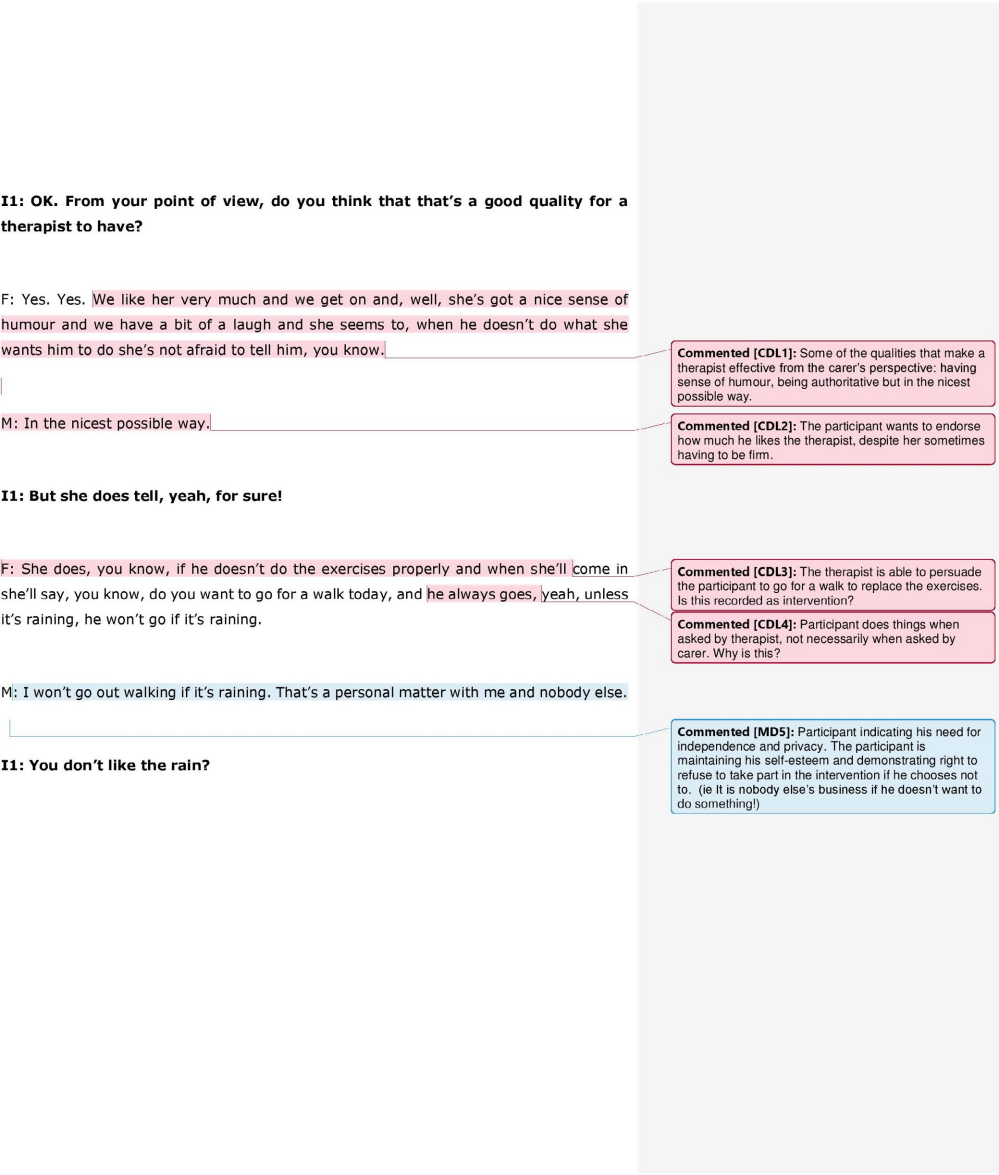
Excerpt of interview transcripts, with annotations from the academic (CDL) and lay researcher (MD)

All the transcripts were analysed through inductive content analysis. The academic researcher underlined relevant pieces of text and wrote coding labels/ideas for each on the margin of each co‐research interview transcript. The co‐research interview transcript files were also sent to lay researchers, who read them and annotated their comments next to the text, independently of the academic researcher's annotations. Although some of the themes identified in the transcripts by the academic and lay researchers were in common, the annotations of lay researchers were instrumental in identifying further aspects relevant to the experience of dementia. In the example showed in Figure 2, for example, the layresearcher identified the themes of independence and privacy as central to the experience of physical activity in participants with dementia.

Once merged, the academic's annotations (from both the co‐research and non‐co‐research interviews) and lay researchers’ annotations (from co‐research interviews) were used by the academic researcher to generate a tentative code book, including themes and subthemes emerging from the transcripts and their operational definitions. The tentative code book was passed to the lay researchers, who gave their feedback, which was used to aid construct refinement. For example, the construct ‘*capability*’ was expanded to include MG’s observation that '*chronic conditions, such as arthritis, heart problems and stroke, might compound upon and mitigate capability*'. The codebook will be used by the lay researchers, who will code two of the interview transcripts each. Inter‐rater reliability between the academic and lay researchers will be tested through Cohen's Kappa coefficient.^32^

#### 2.1.5 Writing and sharing impact

The lay researchers were involved as co‐authors in all study outputs, such as the present paper and the process evaluation protocol.^25^ They will also be co‐authors of the upcoming main process evaluation report and a motivation paper, reporting on aspects affecting participants’ motivation to engage in the programme. The co‐researchers will schedule a visit to the participants to present study findings, so that they can give feedback, once the PrAISED trial is over (ie after month 12). The lay researchers co‐presented research outputs with the academic researcher in seminars and lectures at the University of Nottingham, and a poster on the experience of co‐research at the 2019 Alzheimer Europe Conference in The Hague, Netherlands.

#### 2.1.6 Data collection

All three co‐research team members annotated reflections of their experience of co‐researching in personal diaries, throughout the collaboration. Reflective writing has become established as a method in its own right, as a data source and a key element of qualitative research.^33^ It enables novel perspectives and insight on experience to emerge, which contributes to understanding and learning about practice.^34^, ^35^ Scanlon et al (2002:137)^36^ contend that ‘reflection enables practitioners to tap into knowledge gained through experiences’.

The co‐research team agreed not to use any pre‐imposed template to record their reflections, as this would allow the co‐researchers’ subjective experience to emerge more easily. However, to maintain consistency between the sources, each contributor received guidance to reflect around three main areas of co‐research: methodological issues, benefits, and challenges (as per study aim). It was agreed within the co‐research team, that the personal reflections would be annotated as soon as possible after each qualitative interview with participants, to ensure retentions of fresh memories from the session. A summary of the reflective pieces compiled is featured in this study (see results).

#### 2.1.7 Data analysis and model generation

Once compiled, the individual reflections from each co‐researcher were collated by the academic researcher and stored safely in a password‐protected computer to ensure data confidentiality. At the end of the co‐research experience, the co‐research team joined in a three‐hour session held in one of the lay researchers’ home (MG) to analyse the data and co‐produce a model for good practice in co‐research with carers of people with dementia.

In this session, the co‐researchers individually identified and extrapolated from their own diaries aspects they deemed as relevant for good practice (Table 1). In a team effort, the co‐researchers then aggregated similar aspects reported by the different co‐researchers and generated overall principles on how researchers should address these aspects to pursue good practice in co‐research.

**Table 1:** Identification of relevant aspects in co‐research and how these can be addressed in practice

Co‐researcher	Aspects identified as relevant through the co‐researchers’ reflective diaries	Overall principle (ie how to address the aspects). The academic researchers should…
MD (lay researcher)	The lay researchers feeling worthy/motivated	Adopt a non‐tokenistic approach, build rapport/ foster trusting relationships, give back to lay researchers, keep lay researchers in the loop
	Developing questions that are understood/acceptable to participants	Establish research roles (to each their won expertise), involve lay researchers as co‐authors in research outputs (ie publications and dissemination), give up control on research
	The lay researchers feeling confident to be of value to the study	Provide iterative training
	Managing sensitive situations with participants	Ensure safety of all involved
	Treating participants with dignity and respect	Select lay researchers who have the right skillset
	Avoiding a totally academic viewpoint (eg bringing out issues that might be withheld by participants)	Invite lay researchers to analyse data independently of academic researcher
	Reflecting on strength and weaknesses of co‐research through data analysis	Use transcripts of interviews and keep a reflective diary to derive learning points
	The lay researchers opening up too much/deflecting from interview purpose	Ensure that relevant info is shared and collected during the interview
	Identifying areas of relevance that may pass unnoticed to academics	Invite lay researchers to analyse data independently of academic researcher
	Eliciting genuine, non‐deferential responses from participants	Promote an equitable interview session where all involved are comfortable
	The lay researchers developing the confidence in academic meetings to use their own lived experiences to support or challenge research	Have an open mind and be prepared to step out of your comfort zone
	The lay researchers being able to bring out emotional thoughts, quality of life, and daily mundane problems that could be overlooked, if an academic is concentrating on other aspects and outcomes	Give up control on research
MG (lay‐researcher)	The lay researchers approaching the interviews understanding the study	Select lay researchers who have previous experience of PPI in research
	Ensuring the lay researcher is fully confident to make their contribution and meet the challenges of co‐research	Ensure resources are in place (eg for training, costs)
	Establishing a connection with the participants and using empathy and understanding to widen and deepen the participants’ experience	Select lay researchers who have the right skillset
	Creating an initial bond of trust with participants	Build rapport/trusting relationships
	Helping carers to open up	Promote an equitable interview session where all involved are comfortable
	Helping lay researcher further confidence in their skills and affirming their underlying motivation	Travel together to interviews (eg to brief and debrief)
	The lay researchers committing time away from home	Allow extra time for planning, ensure resources for lay researchers are in place
	Requiring great skills, experience and knowledge on the part of the lay researchers, particularly during the interview	Provide iterative training
	Academic team having an underpinning expectation of lay researchers meeting very high standards	Provide iterative training, have an open mind, ensure resources for lay researchers are in place
	Research participants’ investment in the study deserving a highly skilled interviewer	Provide iterative training, ensure resources for lay researchers are in place, promote interview session where all involved are comfortable
	Co‐research affecting the lay researcher emotionally	Ensure safety of all involved, ensure resources for lay researchers are in place, travel together to interviews (eg to allow time for debriefing/processing emotions)
	Academics undermining contributions of lay researchers in the research agenda, as they do not conform to ‘rigorous’ academic models	Adopt a non‐tokenistic approach, give back to lay researchers, have an open mind, step out of your comfort zone (eg challenge academic culture)
CDL (academic researcher)	The lay researchers helping to make the research documents and the topic guide more language‐appropriate to the participants	Establish research roles and expertise, give up control on research
	The lay researchers helping to identify areas which might be especially relevant to the participants’ experience	Establish research roles and expertise, give up control on research
	Ensuring an open and equal relationship between participants and interviewers	Promote an equitable interview session where all involved are comfortable
	Showing the participants that the research team really values the inclusion and empowerment of people with lived experience in research	Adopt a non‐tokenistic approach
	Establishing an empathic bond with the participants	Select lay researchers based on skills and experience
	The lay researchers helping to make the session less formal, thus creating a relaxed atmosphere	Build rapport/foster trusting relationships
	The co‐research team having a good demographic balance, which well suits the participants’ diverse range of characteristics	Select lay researchers based on skills and experience
	The co‐research team having a mix of personalities. which enhances data collection, as the participants were more likely to find a type of personality they were better matched with	Select lay researchers based on skills and experience
	The lay researcher having greater ‘situational sensitivity’	Give up control on research
	Grasping different nuances of the interviews in data analysis	Invite lay researchers to analyse data independently of academic researcher, involve lay researchers as co‐authors in research outputs (ie publications and dissemination), give up control on research
	Containing academic researcher's bias in data analysis	Invite lay researchers to analyse data independently of academic researcher, involve lay researchers as co‐authors in research outputs (ie publications and dissemination), give up control on research
	Delaying data collection, as extra time is needed to agree on appointment dates	Get guidance from experts
	The lay researchers empathising and relating more easily with their own peers (ie the carer‐participants) than with participant with dementia	Promote an equitable interview session where all involved are comfortable
	Risk of carers revealing information to lay researchers out of the formal interview session	Ensure that relevant info is shared and collected during the interview, provide iterative training
	Agreeing on rigorous research protocols within the co‐research team, prior to contact with the research participants	Get guidance from experts, set up plans for collaboration

The co‐research team then mapped out these principles in the previously referenced diagram illustrating different stages of the research cycle^17^ and derived an ad hoc Research Cycle Model outlining the principles for good practice.

## 3 RESULTS

### 3.1 The lay researchers’ personal reflections

#### 3.1.1 Lay researchers MD reported

Being involved with the study through the whole process, contributing knowledge gained from lived experiences, was fulfilling and made me feel I had, in a small way, given back to the community. Working together with my PPI colleague and an academic who is motivating and committed to the ethos of lay research was fulfilling and gave us a feeling of worth and motivation.

The training sessions were very helpful. At the briefing meeting, attended by the academic researcher, my fellow layresearcher and myself, we discussed the structure of the interviews, and decided who would ask which questions and what was expected of us. As lay researchers, we were able to give input into how the questions should be delivered, to ensure these would be understood and acceptable to both participants and carers. When I left this meeting I felt far more confident that I could be of value to the research project.

In the context of data collection, meeting participants and carers in their homes to discuss personal information required a great deal of experience in managing sensitive situations. A natural ability to relate and empathize with a wide sociocultural base was also required, to ensure participants and carers were treated with dignity and respect. Prior to retiring, I worked in the National Health Service (NHS) as an Administration Manager and had daily contact with end‐of‐life patients. In the interview sessions, I felt I was able to use the personal skills learned in my working life to empathize with participants and carers on a similar level. I was able to come alongside the participants and carers on a day‐to‐day level, rather than from an academic viewpoint, bringing out problems and worries that perhaps could be withheld, if people were overawed by academic professionals. It was interesting to notice that the empathy created between the participant, the carer, and myself enabled them to relax and share relevant thoughts that they may have felt a professional would not be interested in.

Undertaking the review of transcripts following the interviews was a useful exercise in collating thoughts and reflecting on the strengths and weaknesses of using lay researchers. I was concerned that I had talked about my own experiences too much and had deflected from the purpose of the interviews. However, on reading the transcripts, it was apparent that my personal thoughts had a positive effect and empowered the carer to speak about worries that may otherwise have been withheld. It was also interesting to reflect on the changing mood of the participant and carer, identify areas of stress, anxiety or strain on the carer trying to manage difficult situations and understand the importance of social contact and family support. Many of these findings, I feel, were drawn out during casual comments from the layresearcher.

By default, research participants could still view healthcare professionals and academics as being on a different level, particularly older people, who may have been brought up not to challenge their authority. The situation could change, as the younger generations do not have the same deferential attitudes. At present, however, while academics may have lived experiences and an understanding of the participants’ perspectives, it will not change how the participants, in return, view the professionals. This could be reflected in their responses to the interviews, if a lay person was not there.

My involvement presented with some challenges too. The experience my PPI colleague and I brought from careers outside the confines of academia sometimes clashed with the academics’ views and needs. For example, in relation to participant‐fronting documents, I found it was difficult to balance academic language requirements while also ensuring understandable wording for participants. Having the confidence in academic meetings to use my own lived experiences to support or challenge research also presented with challenges. Sitting with academics at research team meetings was at times daunting. When the academics used language unfamiliar to lay people, I felt inhibited to speak out. This might have limited the added value of hearing my views as a lay person.

The main lessons I have learned from the experience is how important it is in people‐centred research that the academic researcher is open to using lay people and is not dismissive of their views. Being able to identify with participants and carers is not only an intellectual process. Lay researchers are able to bring out emotional thoughts, quality of life, and daily mundane problems that could be overlooked, if an academic is concentrating on other aspects and outcomes. Co‐research is still in its infancy stage, protocols and models need to be created to ensure good practice. The present study, which I and my PPI colleague co‐authored, represents a step in the direction of improving participation in research for people with dementia and their families.

#### 3.1.2 Lay researchers MG added

This has been the most affirming and positive piece of work that I have had the pleasure to be part of, in the 10 years that I have given to PPI, primarily in dementia studies.

Approaching the new experience of process evaluation and co‐researching, over the previous 3 years, MD and I had invested much time and energy and brought our lived experiences together with our knowledge and skills to the PRAISED RCT. We had been an integral part of development of PrAISED and became very familiar with its aims and procedures. Together with helping write the participant‐facing material, my husband and I had modelled as participant and carer for the publicity material and exercises. All this meant that I could approach the interviews confident in my understanding of the study and committed to its success. When we met with the academic researcher, who embraced our roles and ensured we were fully confident to make this further contribution, we knew we could positively meet the challenge. All this enabled us to establish a connection with the participants, and this was clearly such a strong factor in ensuring the interviews were both structured as planned, but still allowed them to evolve organically, as our empathy and understanding helped widen and deepen the experience.

My introduction to the participants as a layresearcher was made easier, as a photograph of me and my husbands fronted the large PrAISED work folder that each participant had been given on their involvement in the study. I think this further helped to lower barriers, as my photograph had practically been with the participant and carer for months. This seemed to create an initial bond of trust, which became a significant part of the interview process. While the physical voices of the carers were heard more often, their inner thoughts and feelings were more often than not put to one side. The carers tended to prioritize reporting of the participants’ needs and their experiences. Therefore, I felt that my responses as a carer with lived experience in a small way helped to draw out the reality of the impact of the dementia on their relationships and lives. Therefore, as a team, we gained a more comprehensive picture of their involvement in the study and the significant changes it brought in their daily living.

The opportunity to debrief with the academic researcher, who brought his professional capacity as a psychologist into every discussion, was a new experience for me and gave me further confidence in my skills. This is rarely if ever given to PPI, but I felt it was vital and highly valuable. It contributed to my experience of PPI working at its very best. It affirmed my underlying motivation and belief that what we lay researchers do make a difference.

There were some practicalities involved in co‐research that might pose a challenge to other PPI members wishing to undertake the role of lay researcher. For example, travelling to participants’ homes and conducting the interviews meant that I had to commit time away from home. Co‐research in this study required great skills on the part of the lay researchers, particularly during the interview. With very limited experience in process evaluation, I and my fellow PPI colleague worked as equals with a highly trained academic researcher. It has to be acknowledged that, by the very nature of being volunteers, the level of skills, experience, and knowledge required for this role might not be met by other PPI members.

The academic team had an underpinning expectation of us lay researchers meeting very high standards. Similarly, the research participants’ investment in the study deserved a highly skilled interviewer to enable them to openly talk about their experiences. These feelings put a lot of pressure on me. The preparation, training, and debriefing provided by the academic researcher were key in boosting my confidence to undertake the role. However, as my life‐experiences paralleled those of the participants, unlike the professionals, I could not see the interview session purely as an academic process. This made co‐researching such a worthwhile part of my journey, but it also affected me emotionally. Retrospectively, I think I should have asked for further support from the academic researcher to work this through.

I think it must be recognized that, given the emotional investment that lay researchers may have in the study, this might have an impact on the interview session. These difficulties can only be overcome through considerable training, in‐depth support, and trust. While these were embedded in the co‐research team, I think that a change of culture is still required in the academic environment at large, to embrace the added value that lay researchers may bring to research studies. At times, I felt that my contributions in the research agenda were undermined, as they did not conform to ‘rigorous’ academic models. The lack of recognition of what true collaboration in research means made me more guarded to undertake some of the co‐research tasks and presented as a challenge for me to work through personally, also at the level of professional rapport with members of the academia.

In conclusion, the experience for me has been very powerful. To meet, at last, with those taking part in the study felt like truly being embedded in the research process. It gave me further insight into others’ lived experiences, enabling me to prepare for a future, which could see me on a parallel journey.

### 3.2 The academic researcher's personal reflections

Involving the lay researchers in the PrAISED process evaluation yielded numerous benefits. Their input was invaluable in making the research documents and the topic guide more language‐appropriate to the participants. The lay researchers also helped to identify areas which might be especially relevant to the participants’ experience of the PrAISED RCT. It was felt that the original topic guide was lengthy and that a long interview session might cause fatigue in the participants. The lay researchers’ input was invaluable in selecting the most relevant questions (eg Improved quality of life through exercise), which were prioritized during the interview session, over those which may yield less insightful responses (eg intervention characteristics).

The benefits of co‐research were also evident in the context of data collection. In this respect, having collected half of the data independently, I was able, by comparison to better identify the added values of co‐research. Being the interview semi‐structured, and thus guided by a pre‐defined topic guide, there were not any differences in relation to the quality or quantity of the data collected. However, the presence of the lay researchers contributed to diffuse tension, thus creating a lighter atmosphere. For example, at the beginning of the interview, the initial introduction of the layresearcher as someone having experience of being a carer of a person with dementia was instrumental in setting the ground for an open and equal relationship between participants and interviewers. The layresearcher also gave a ‘human face’ to PrAISED, showing the participants that the research team really values the inclusion and empowerment of people with lived experience in research. Given their lived‐experience with dementia, the layresearcher was also able to establish an empathic bond with the participant. The carers seemed at ease opening up with someone experiencing a similar journey in dementia and the informality of the situation welcomed the use of humour, which further created a relaxed atmosphere.

Being the co‐research team made up of a younger academic male researcher and an older female layresearcher, the research team had a good demographic balance, which well suited the participants’ diverse range of characteristics. It was found that the participants could relate well (and open up) with the one researcher who had similar age and background, given the shared life‐experiences. A mixed‐gender research team was also a valuable asset with the couples who adopted strict gender roles. It was noticed that female participants would more often keep eye contact with the female researcher, while the male participants would find it easier to relate with the male researcher. It was also observed that a mix of personalities in the research team was helpful to enhance data collection, as the participants were more likely to find a type of personality they were better matched with and open up more easily about their experience.

Other than personal characteristics, the lay researchers brought added value to the interview process, given their lived experience with dementia. They displayed great ‘situational sensitivity’, which is ‘consideration of the interest and vulnerability of the particular participant, rather than application of general rational principles’.^37^ For example, knowing first‐hand the sensitivity of terminology, though this was not established as a ground rule during the preparatory work leading to data gathering, the lay researchers avoided using the term ‘dementia’, unless it was first mentioned by the carer or the participant during the interview.

In terms of data analysis, the differences between the two different configurations (ie with and without the input of lay researchers) were more marked. Having different background and experiences, the lay researchers’ transcript annotations grasped different nuances of the interviews. For example, MD tended to focus on the use of language and its underlying meaning, while MG on aspects related to quality of life and social networking. The inclusion of the perspective of lay researchers in the generation of themes and subthemes was therefore instrumental to contain academic researcher's bias and allowed validation of process evaluation data with people with lived experience with dementia.

There were also some challenges experienced in co‐research. Undertaking co‐research might potentially delay data collection, as extra time is needed to agree on appointment dates, which are suitable to all those involved in the process. While co‐delivering the interview with lay researchers aims to make the process more equitable for all research participants, it must be recognised that when lay researchers are carers, they might more easily empathize and relate with their own peers (ie the carer‐participants). In such scenario, the participant with dementia might still perceive the interview as disempowering. The empathic bond between the lay researcher and the carer participant might also have repercussions on data collection. It was noticed that some carers tended to confide in the lay researcher out of the formal interview session, revealing information that were relevant for the study in confidence to the lay researcher. This suggests the importance of rigorous research protocols to be agreed within the co‐research team, prior to contact with the research participants.

### 3.3 Generation of a model for good practice

**Figure 3:**
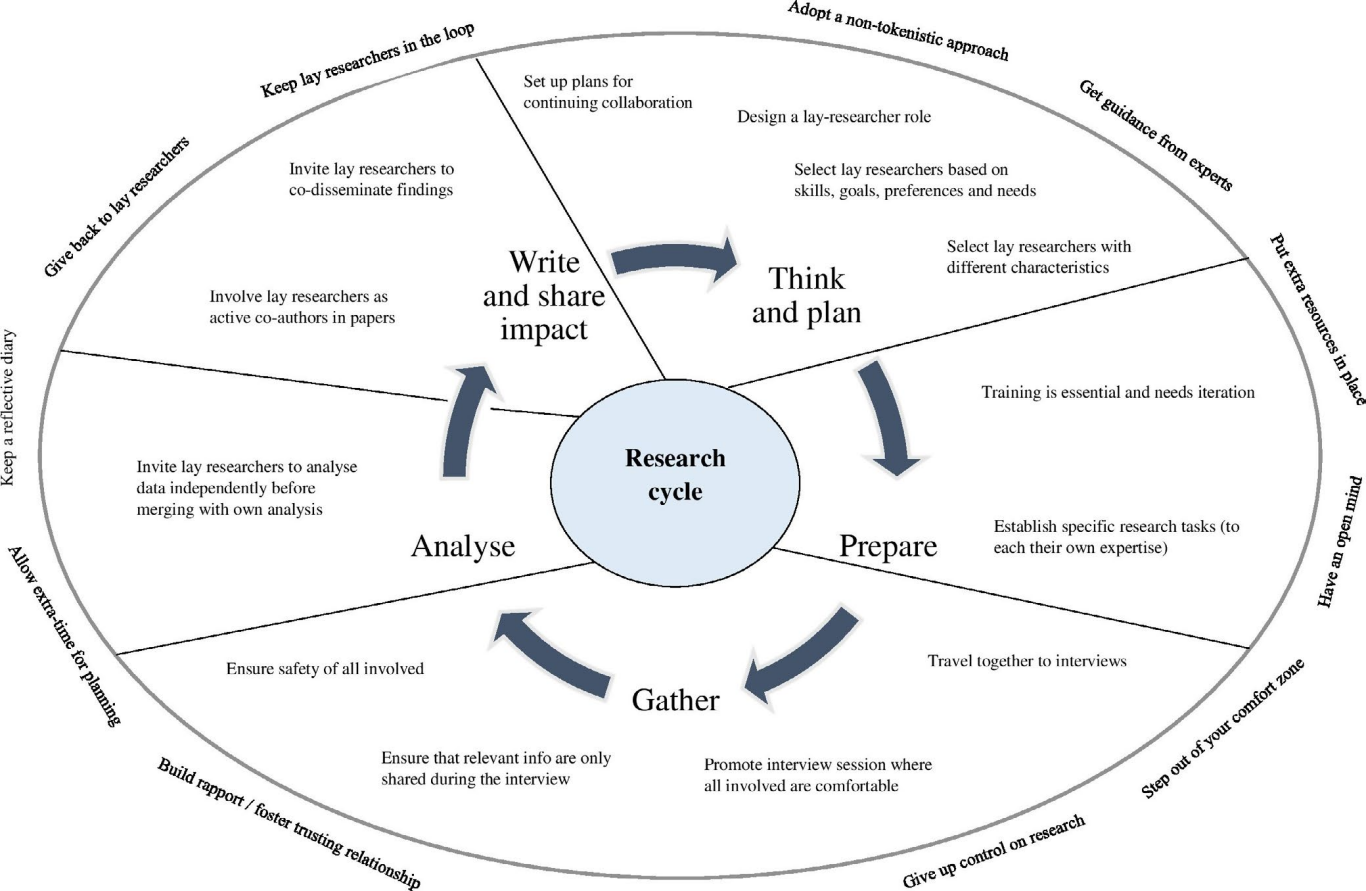
A model for good practice in co‐research with carers of people with dementia

The model for good practice in research with carers of people with dementia (Figure 3) features research‐stage‐specific and overall principles. While the former apply specifically to co‐researching with carers of people with dementia, we feel that the latter represent transferable information which can be used in co‐research with other members of the general public (ie without experience of dementia).

In relation to the research‐stage‐specific guidelines, the ‘think and plan’ stage requires the set‐up of plans for long‐term collaboration with lay researchers (during and after the research cycle) early on in the research^38^ and an appropriate selection of the lay researchers. Ideally, the layresearcher role should be co‐designed with PPI members who wish to be involved. In order to facilitate effective and meaningful contribution in the research process, attention should be dedicated to both the lay researchers’ aspirations, preferences, and goals to be obtained from involvement and to the skills they need to fulfil their research role. It is also ideal to select lay researchers with different skillsets, background, experience from each other, so that each of them can contribute uniquely to the process. Although separate ethical approval was not a requirement for co‐research in this study, ethical and governance demands may vary across different institutions, as reported in the literature.^15^, ^39^ Academic researchers should therefore work proactively to ensure that approvals (eg letters of access for lay researchers to have contact with research participants recruited through the NHS) are in place when involving lay researchers. In the ‘prepare’ stage, the model emphasizes the importance of training offered to lay researchers, which needs to be on‐going throughout the project, based on emerging needs. At this stage, it is also essential for good working relationships to discuss, negotiate, and establish clear research roles.

In the ‘gather’ stage, the co‐research team members should share commuting to the location of the data collection, which will promote bonding and allow pre‐session preparation and post‐session discussion. This may also resolve transportation issues that the lay researchers may have to reach the interview locations. To ensure effective data collection, all those involved in the session should be made comfortable. In order to make the interview process equitable for the participant with dementia, the team should send photos of the researchers in advance, so that the participant can get familiar before the session. The photos could also help the participants with memory impairment to remember the co‐researchers in the follow‐up visit. In the context of a co‐research interview, four people might be present. Given the ‘expertise’ of co‐researchers, the participants with dementia and their carers may be in a disadvantaged position, as they might be less articulate and feel less confident to voice their views. This potential power differential may require great skills of interaction management on the part of all researchers, to ensure equitability in the interview process.^15^ Again, this highlights the importance of training.

Training is also crucial to ensure data integrity. An important methodological issue we encountered is the moral imperative to give support and advice that lay researchers may experience when hearing the participants’ difficulties. This may breach boundaries between research interview and intervention and have an impact on data integrity. In the lack of appropriate training, there is a risk of actively influencing responses, as opposed to merely elicit them.

During the interview process, it is also crucial to ensure the physical and emotional wellbeing of the layresearcher, who should always be supported by the academic researcher. As found in previous studies, when hearing the difficulties of the participants, the lay researchers might reflect on their own situations and become emotionally stressed.^15^ Therefore, strategies should be in place for lay researchers to discuss any concerns/ emotional issues. Ideally, a specific distress protocol should be in place to provide verbal and written support to lay researchers.

In the ‘analyse’ stage, the model emphasizes the importance of lay researchers interpreting/analysing data independently of the academic researcher, before results of the analysis process are merged. This will ensure different perspectives and their own voices to be reflected in the research outputs. In line with previous studies,^39^ in the ‘write and share impact’ stage, lay researchers should contribute as equals in writing reports and dissemination materials (as active co‐authors, by writing their own sections) and in presenting research outputs at conferences, seminars, and talks.

In relation to the overarching action points (ie applicable to all stages of the research cycle), the academic researchers should strive for non‐tokenistic and meaningful collaboration in all research stages. In order to engage effectively in co‐research, they can seek help and guidance from experienced researchers in the field and/or university resources. The academic team should allocate extra resources in place to pursue good practice in co‐research, including extra funds, time, and staff.^15^ In the context of a large RCT, financial and staff resources were such that co‐research could be adequately funded, even though it had not been considered/costed in the original project proposal/ protocol. However, given its resource requirements, academics willing to undertake co‐research should calculate costings from the initial stages of the project application.

Throughout the co‐research experience, it is crucial to cultivate good rapport within the team, and foster trusting relationships. This could be facilitated by members spending time away from work together. The whole experience of co‐research should ideally be recorded in reflective diaries, which can be used not only as key data sources, to advance good practice, but also to promote personal and professional development. Finally, as also reported in previous studies,^29^ the wider research team should give back to lay researchers (in the form of financial compensation and/ or professional and personal development), to acknowledge their invaluable contribution to research. Involve guidelines^27^ ensure adequate compensation, so that it can be costed appropriately. In keeping the lay researchers motivated to actively contribute through the research project, as also found in previous studies,^29^ the academic team should set up systems and official channels (eg monthly project meetings) to keep them updated about the project.

## 4 DISCUSSION

This methodology paper aimed to add to the existing knowledge of good practice in PPI, by reporting on an experiment of co‐research with carers of people with dementia in the context of a large RCT (PrAISED) and by developing, based on results, a model for good practice (Figure 3). In line with the current PPI standards,^8^, ^28^ two PPI members with experience of caring for someone with dementia and an academic researcher collaborated as equals in all stages of the research cycle, including designing the study protocol, developing the topic guide, collecting and analysing data, and disseminating research findings. The novelty of this work, compared to the existing literature is that, by having a dedicated section in the paper where to report their reflections on the process of co‐research, the lay and academic researchers shared equal power as contributors in results dissemination. Involving lay researchers as co‐authors, we feel, is in line with full and meaningful involvement in research.

The study is characterized by certain strengths and limitations. In some previous experiments of co‐research with carers of people with dementia, the lay members were involved at later stages of the research cycle (eg after the interview topic guide had received ethical approval).^15^ A strength of this study is that the lay researchers were involved since the development phase of the process evaluation, giving carers of people with dementia an active research role throughout the whole research cycle. Although we only involved two lay researchers, the experience they accumulated through previous PPI roles, their knowledge of the PrAISED RCT and the interview skills that they built through their past working experience greatly contributed to gather enhanced research data.

For future implications for research, while there is an increasing demand for PPI, in fact a requirement for study grants, a culture change in academia is still required to combat tokenism and ensure that members of the public are not merely used as ‘assets’ to gives PPI accreditation to research studies. In collaborating with the lay researchers, an open mind is crucial, and the academic researchers should be ready to challenge traditional views on research, step out of their comfort zones, and cede control over the research process. This may require specific training or a change of culture. Despite advancements, as reported in previous studies,^39^ further resources should be available within research projects to enhance the skills of academic researchers to effectively engage in PPI through training.

Further resources are also required to recruit PPI members, which are currently involved in research mostly through snowball sampling (ie PPI volunteers already involved in research proactively recruiting their friends, colleagues, and community group members). This would enable recruitment of diverse public and patient contributors. Finally, resources need to be in place to fully support PPI members’ needs when undertaking research roles. Despite the lack of training and little financial compensation, PPI members have been increasingly asked to undertake research roles that require a high level of skill and knowledge.

In conclusion, if academic institutions are to meet the challenges of more effective and meaningful PPI in research, there is a need for more structured support of academic and lay researchers. In this respect, the future of PPI in research lies in the accumulation of knowledge granted by studies such as this one. This study showed that co‐research with carers of people with dementia may yield benefits, both on the personal and professional levels of those involved and in terms of research outputs. There are certain challenges and practicalities that require careful consideration in order to make the involvement of lay researchers meaningful and effective, which can be addressed with extra planning and resources and the sustained commitment of university institutions.

## CONFLICT OF INTEREST

None to declare.

## Data Availability

Data are available from the authors, on request.

## Appendix 1

**Table:**

No. Item	Guide questions/description	Reported on
Domain 1: Research team and reflexivity		
Personal Characteristics		
1. Inter viewer/facilitator	Which author/s conducted the interview or focus group?	Page 4
2. Credentials	What were the researcher's credentials? eg PhD, MD	Page 4
3. Occupation	What was their occupation at the time of the study?	Page 4
4. Gender	Was the researcher male or female?	Page 4
5. Experience and training	What experience or training did the researcher have?	Page 4
Relationship with participants		
6. Relationship established	Was a relationship established prior to study commencement?	Page 7
7. Participant knowledge of the interviewer	What did the participants know about the researcher? eg personal goals, reasons for doing the research	Page 7
8. Interviewer characteristics	What characteristics were reported about the inter viewer/facilitator? eg Bias, assumptions, reasons, and interests in the research topic	Page 7
Domain 2: study design		
Theoretical framework		
9. Methodological orientation and Theory	What methodological orientation was stated to underpin the study? eg grounded theory, discourse analysis, ethnography, phenomenology, content analysis	Page 7
Participant selection		
10. Sampling	How were participants selected? eg purposive, convenience, consecutive, snowball	Page 7
11. Method of approach	How were participants approached? eg face‐to‐face, telephone, mail, email	Page 7
12. Sample size	How many participants were in the study?	Page 7
13. Non‐participation	How many people refused to participate or dropped out? Reasons?	Page 7
Setting		
14. Setting of data collection	Where was the data collected? eg home, clinic, workplace	Page 7
15. Presence of non‐participants	Was anyone else present besides the participants and researchers?	Page 7
16. Description of sample	What are the important characteristics of the sample? eg demographic data, date	Page 7
Data collection		
17. Interview guide	Were questions, prompts, guides provided by the authors? Was it pilot tested?	Appendix 2
18. Repeat interviews	Were repeat inter views carried out? If yes, how many?	Page 7
19. Audio/visual recording	Did the research use audio or visual recording to collect the data?	Page 7
20. Field notes	Were field notes made during and/or after the interview or focus group?	Page 7
21. Duration	What was the duration of the inter views or focus group?	Page 7
22. Data saturation	Was data saturation discussed?	Page 7
23. Transcripts returned	Were transcripts returned to participants for comment and/or correction?	Page 7
Domain 3: analysis and findings		
Data analysis		
24. Number of data coders	How many data coders coded the data?	Pages 8
25. Description of the coding tree	Did authors provide a description of the coding tree?	Appendix 3
26. Derivation of themes	Were themes identified in advance or derived from the data?	Page 8
27. Software	What software, if applicable, was used to manage the data?	Page 8
28. Participant checking	Did participants provide feedback on the findings?	Page 8
Reporting		
29. Quotations presented	Were participant quotations presented to illustrate the themes/findings? Was each quotation identified? eg participant number	*
30. Data and findings consistent	Was there consistency between the data presented and the findings?	*
31. Clarity of major themes	Were major themes clearly presented in the findings?	*
32. Clarity of minor themes	Is there a description of diverse cases or discussion of minor themes?	*

* These items are not applicable, as this is not the study reporting findings from the process evaluation.

## Appendix 2

### Topic guide for qualitative interviews

NB ‐ the following questions are suggestions and prompts ‐ some answers may be anticipated earlier in the discussion and others turn out to be not relevant. The interviewee may also raise additional topics and issues which they feel are particularly relevant and these should be followed up in the discussion.

#### Pre‐interview

• Researcher introduces himself and engages in small talk to break the ice with participant (eg give thanks for being invited over, gives compliments about the home, and asks how the person is doing on the day).
• Researcher explains his professional role and the purpose of the visit
• Researcher goes through the Information Sheet with the participant. The following will be clearly explained:
  1 The interview will be audio‐recorded to have an accurate record of what was said
  2 Anything mentioned during the interview is confidential and no one except members of the PrAISED research team will know what was said
  3 In using any information in a report, the participant's anonymity will be maintained
  4 Participation is totally voluntary
  5 The participant can withdraw at any time and the research team can use the information collected thus far, unless the participant specifically withdraws consent for this.
• Researcher asks if participant has any concerns/questions/doubts.
• Researcher seeks informed consent
• Researcher asks participant if they are comfortable being interviewed alone or they prefer the presence of a carer during the process

#### Interview

##### General questions

1. Do you feel that being involved in the study has been beneficial?
2. If so, what are the positive results of the activities?
3. Have you experienced any negative effects of the activities?
4. Do you think the programme has enabled you to enjoy more your daily activities?
5. I would like to start by asking your views around exercise…

##### Personal beliefs

• How important do you think being active is to help people stay healthy?
• How important do you think being active is to help people stay independent?

##### Motivation

• Why did you decide to take part in the programme?
• Were you encouraged by anyone to take part or was it your own choice?
• What helps you keep going with the programme?
• On a scale from 1 to 10, how much do you feel you want to continue with the Activities, once the programme has finished?

##### Autonomy and control

• Is it important for you to decide what you do or do you prefer to leave it to others?
• (If yes to previous question), how much have you been able to make those decisions?
• How could we make you feel more involved?

##### Intervention characteristics

• Does the programme of physical activities suit your needs and preferences?
• What part of the programme of physical activities do you like the most?
• What part do you like the least and how could this be improved?

##### Self‐efficacy and emotional support

• Do you feel you are able to do the activities as well as you would expect?
• Do you have any concerns or anxiety about taking part in the programme/doing the activities?
• Did you receive encouragement and support from your therapist(s) and carer(s)?
• Is there anything that would help you feel more confident to do the activities?

##### Support (Practical)

• Do the therapists give you practical support? For example, do they show you how to do the activities, when to do them and where to do them?
• Does your (carer role) give you practical support? For example, does he/she remind you how to do the activities, when to do them and where to do them?
• What could be done to better support you?

##### Independence

• How has the study programme affected you? (eg on your health and activity)
• Has it given you greater independence?
• Have you noticed a change in your quality of life?
• Are there any activities you would like to be able to do that are not part of the programme?

##### Expectations

• Have you any personal goals you would like to achieve from the study?
• If yes, what goals are you looking to gain?
• Do you think you can achieve these goals and do you need support to do this?

### Final remarks

• Any final thoughts and feedback on the programme?
• Would you be happy to meet up again in 3 months’ time to see how you are doing?

## Appendix 3

### Final version of codebook

**Table:**

Theme	Operational definition
Characteristics of the person with dementia	Characteristics of the person affecting behaviour change, which include personality, temperament, and identity
Support	Practical and emotional support from others (eg carer, therapist, society) which affects behaviour change
Expectations/goals	Expectations/goals around the behaviour, including benefits, barriers, and facilitators
Carer(s)	Any aspect, behaviour and attitude of the carer, which mediates behaviour change and maintenance
Progress	Perceived or actual improvement in the person's physical or mental health, following the behaviour
Social opportunity	Social contacts and networking opportunities (or lack thereof) granted through engaging in the behaviour
Self‐efficacy	Confidence in one's ability to execute a given behaviour, including (perceived) physical, cognitive ability, and competence
Capability	One's actual ability to perform a behaviour through essential skills, including (actual) physical, chronic conditions, cognitive ability, competence psychological/personal and social capability
Activity/intervention characteristics	Characteristics of the activity or intervention which influence participants’ engagement in it. They include how much the participant felt they are tailored to their needs, goal, preferences, and aspirations, how helpful, enjoyable, and challenging they are and how they fit into their routine
Autonomy/control	Being causal agents of one's behaviour
Physical infrastructure	Environment and its characteristics, where the behaviour change occurs
Personal history	Personal history of a person, which affects present behaviour change
Information/knowledge	Information and knowledge that the person needs to change their behaviour
Professional	Any aspect, behaviour, and attitude of the professional, which mediates behaviour change and maintenance
Personal beliefs	The self‐regulated mechanisms that the person uses in relation to initiation, adherence, and withdrawal from behaviour change
